# Therapeutic Extracellular Vesicles from Synovial Fibroblast‐Primed MSCs for Osteoarthritis Treatment

**DOI:** 10.1002/adhm.71406

**Published:** 2026-07-06

**Authors:** Seo Jeong Kim, Ji Seob Kim, Min Hee Moon, Jae‐Hyeok Jang, In Sun Hwang, Byung‐Hyun Cha

**Affiliations:** ^1^ Department of Biomedical Systems Science, College of Biomedical Science Kangwon National University Chuncheon‐si Republic of Korea

**Keywords:** cell to cell interaction, extracellular vesicles (EVs), mesenchymal stem cells (MSCs), osteoarthritis (OA), synovial fibroblasts

## Abstract

Mesenchymal stem cells (MSCs) migrate to injured tissues through a homing effect and promote tissue regeneration by secreting paracrine factors and extracellular vesicles (EVs) and interacting with resident cells. MSC‐derived EVs have emerged as promising therapeutic candidates for osteoarthritis (OA) because they carry bioactive molecules, are easily delivered, and exhibit low immunogenicity. In this study, we generated MSCs with altered cell fate by transferring the microenvironment of SW982 human synovial fibroblast‐like cells through direct MSC–synovial fibroblast (SF) interaction and evaluated EVs derived from these cells (miSF‐MSC‐EVs) as a therapeutic strategy for OA. MSCs and SFs were stained with nuclear dyes, co‐cultured for 48 h, and double‐positive cells were isolated by fluorescence‐activated cell sorting. Proteomic and next‐generation sequencing analyses revealed enrichment of miRNAs associated with cell migration, adhesion, and transforming growth factor‐β, Wnt, and PI3K signaling pathways in miSF‐MSC‐EVs. Compared with conventional MSC‐EVs, miSF‐MSC‐EVs enhanced cell proliferation, improved regenerative responses, reduced inflammatory marker expression, and increased anti‐inflammatory marker expression in vitro. Furthermore, miSF‐MSC‐EVs promoted cartilage repair in a mouse OA model, highlighting their potential as a regenerative therapeutic platform for OA treatment.

## Graphical abstract

EVs derived from the microenvironment of SFs transferred MSCs (miSF‐MSC‐EVs) enhanced the expression of miRNAs that regulate cell migration, cell adhesion, and the TGF‐β signaling pathway, demonstrating superior anti‐inflammatory and cartilage regeneration effects compared to conventional MSC‐EVs.

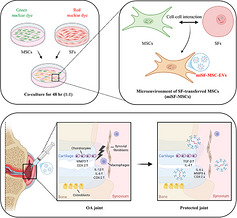

## 1 Introduction

Osteoarthritis (OA) is the most common joint disease in older adults and is characterized by articular cartilage loss, subchondral bone dysfunction, and synovial inflammation [1]. The synovium is a thin, delicate membrane that surrounds articular joints and maintains synovial fluid homeostasis to support smooth movement. It also functions as a barrier and secretes hyaluronic acid and lubricin [2, 3]. Synovitis, accompanied by macrophage and lymphocyte infiltration, is a key contributor to OA pathogenesis [4]. Inflammatory mediators, including interleukin‐1β (IL‐1β) and interleukin‐6 (IL‐6), as well as the cartilage‐degrading enzyme matrix metallopeptidase 3 (MMP3) and the inflammation‐sustaining enzyme cyclooxygenase‐2 (COX‐2), promote cartilage degeneration and OA progression [5, 6]. Patients with synovitis report higher pain levels, suggesting that synovial cells may represent an early therapeutic target [7]. Current management primarily focuses on symptom relief using nonsteroidal anti‐inflammatory drugs (NSAIDs), glucocorticoids, or analgesics, but these interventions provide limited benefit and can cause adverse effects. Moreover, available pharmacologic approaches do not reliably modify disease progression or prevent OA. Given the heterogeneity of OA, effective and personalized therapeutic strategies are needed [8, 9, 10].

Human mesenchymal stem cells (MSCs) contribute to tissue repair by differentiating into multiple lineages, including bone, cartilage, and muscle, and by acting as progenitor cells [11]. In damaged tissues, cytokines, chemokines, and growth factors induce MSC homing and regulate their migration through the circulation [12]. After reaching the injury site, MSCs interact with resident cells through both direct and indirect mechanisms to promote regeneration. Direct cell–cell interactions are particularly important for intercellular communication as they enable transfer of biochemical signals and cellular components via physical contact, including gap junctions and adhesion molecules [13, 14, 15]. These interactions activate signaling pathways that relay information to the nucleus, modulate gene expression, and influence cell fate decisions, including proliferation and differentiation [16, 17]. Accordingly, co‐culture systems that enable direct cell–cell contact provide a useful platform for engineering cell behavior and may enhance the therapeutic efficacy of cell‐based regenerative approaches.

Extracellular vesicles (EVs) have recently attracted considerable attention due to their small size, which facilitates delivery, and their low immunogenicity [18, 19]. EVs are produced by all cell types and are released as lipid bilayer‐enclosed particles that mediate biological processes and intercellular communication by transferring proteins, mRNAs, and miRNAs. EVs span a broad size range (30–5,000 nm) and include exosomes, microvesicles, and apoptotic bodies [20]. As important mediators of intercellular communication, EVs enable the exchange of bioactive cargo between neighboring and distant cells, thereby modulating the tissue microenvironment and cellular responses under both physiological and pathological conditions [21, 22, 23]. They are integral components of the microenvironment, contribute to cellular homeostasis by removing dispensable intracellular material, and participate in survival signaling, apoptosis, and immunomodulation [24, 25]. EVs derived from MSCs exhibit immunomodulatory and paracrine activities that support tissue regeneration [26, 27], and several EV‐associated miRNAs have been implicated in OA therapy [28, 29, 30].

On this basis, we hypothesized that MSCs exposed to the microenvironment of a human synovial fibroblast‐like cell line (SW982) through direct cell–cell interaction (miSF‐transferred MSCs) would acquire distinct properties and that EVs derived from these cells (miSF‐MSC‐EVs) would exhibit enhanced therapeutic activity relative to conventional MSC‐derived EVs (MSC‐EVs). Profiling analyses indicated that miSF‐MSC‐EVs are associated with pathways regulating cell migration, cell adhesion, and key signaling networks. Functionally, miSF‐MSC‐EVs more effectively suppressed inflammatory responses and promoted proliferation in synovial fibroblasts and enhanced cartilage regeneration in a mouse OA model. Together, these findings support miSF‐MSC‐EVs as a potential therapeutic platform for OA.

## 2 Results and Discussion

### 2.1 Microenvironment of Synovial Fibroblast(miSF)‐Transferred MSCs via Cell‐Cell Interaction

Synovitis secondary to cartilage degradation contributes to OA progression [31]. Fibroblast‐like synoviocytes (FLS) produce interleukin‐1β (IL‐1β) and tumor necrosis factor‐α (TNF‐α), which promote cartilage degradation [32], and OA patients with synovitis report higher pain levels [7]. Accordingly, synovial cells represent attractive therapeutic targets for alleviating symptoms and limiting joint structural damage [33].

**FIGURE 1:**
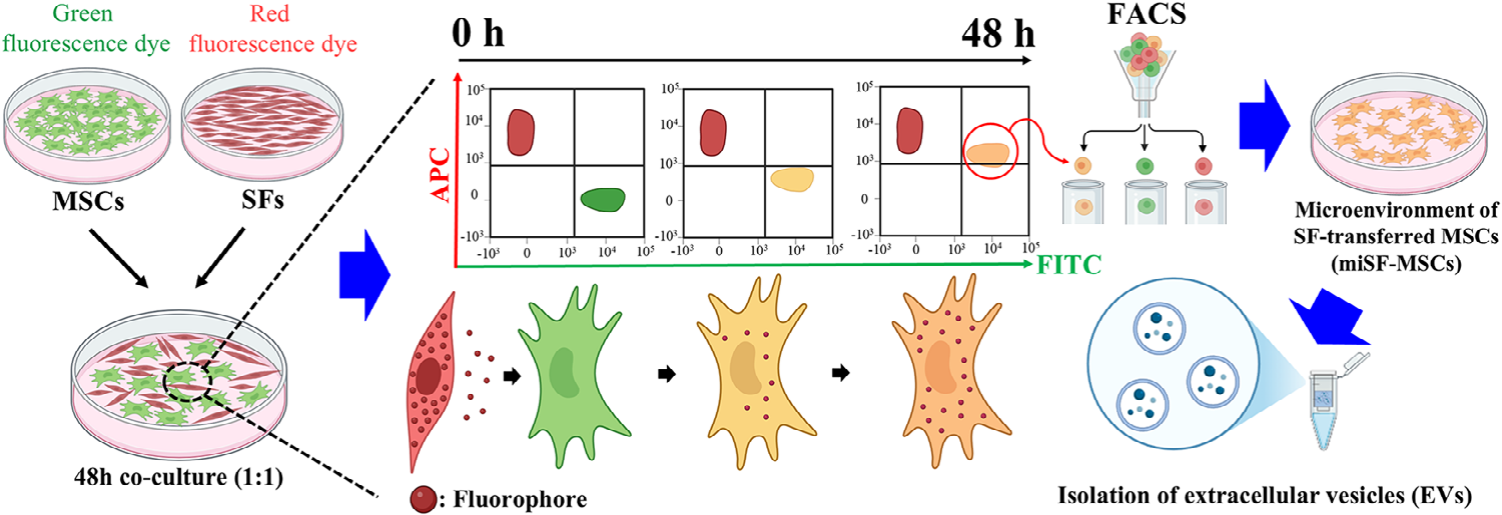
Schematic illustrating the process of generating miSF‐MSCs via co‐culture using FACS. MSCs and SFs were labeled with green and red nuclear dyes, respectively, and co‐cultured at a 1:1 ratio for 48 h. Double‐positive cells, defined as cells acquiring counterpart‐derived fluorescent signals during co‐culture, were sorted as miSF‐MSCs. EVs were then isolated from the sorted miSF‐MSCs.

MSCs exhibit phenotypic plasticity in response to microenvironmental cues [34]. To leverage this property, we co‐cultured MSCs with synovial fibroblasts (SFs) to generate miSF‐transferred MSCs (miSF‐MSCs). During co‐culture, cell–cell interactions enable biochemical exchange between MSCs and SFs, thereby altering MSC fate and gene expression. To obtain miSF‐MSCs, MSC and SF nuclei were labeled with green and red dyes, respectively, and co‐cultured at a 1:1 ratio for 48 h. Cells positive for both green (FITC) and red (APC) fluorescence were then isolated by fluorescence‐activated cell sorting (FACS) (Figure 1).

**FIGURE 2:**
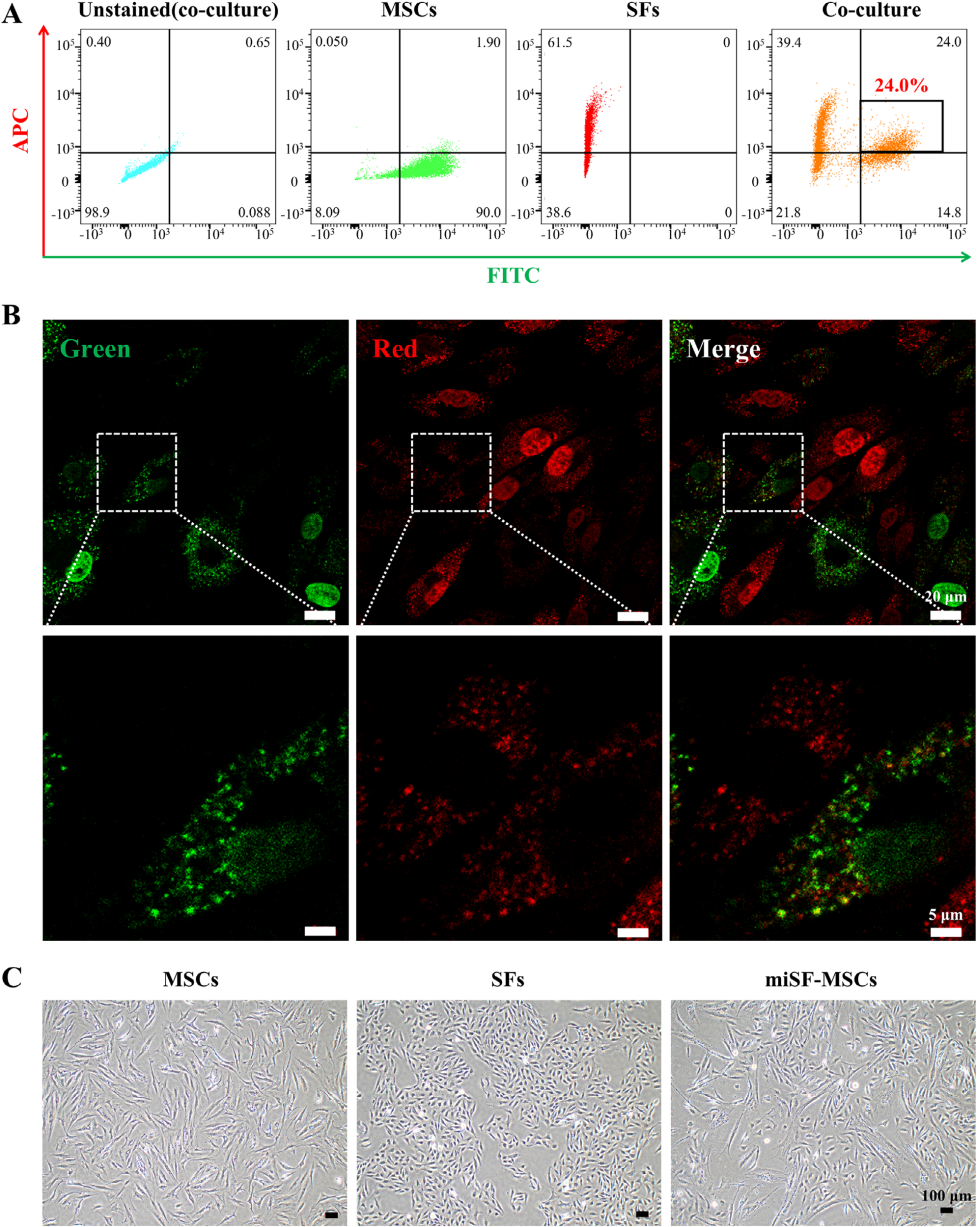
Sorting and morphology of miSF‐MSCs. (A) After co‐culture of MSCs and SFs, the flow cytometry results showed that the sorted miSF‐MSCs were 24.5%. Double‐positive gating was defined with reference to the fluorescence distributions of MSC‐only and SF‐only control populations. (B) After co‐culture for 48 h, interactions between MSCs and SFs were confirmed by CLSM. The length of the scale bars: 20 and 5 µm. (C) Morphology of MSCs, SFs, and miSF‐MSCs. miSF‐MSCs exhibited a morphology both similar to MSCs and SFs. The length of the scale bars: 100 µm.

Double‐positive cells were recovered from the co‐culture group but not from the unstained control, yielding 24.5% of an operationally defined miSF‐MSC population based on the fluorescence distributions of single‐population controls. Flow cytometry indicated a shift in the MSC population after co‐culture, whereas the SF population remained largely unchanged (Figure 2A). In red dye‐labeled SFs, the signal was confined to the nucleus at 24 h but redistributed to the cytosol by 48 h. In an independent experiment, red fluorescence was nuclear at 24 h and predominantly cytosolic at 48 h, consistent with intracellular redistribution and transfer of dye‐associated material to neighboring cells (Figure S1A). Confocal microscopy further confirmed the presence of double‐positive cells in the co‐culture group, with mixed green and red cytosolic signals, supporting material exchange through direct cell–cell interaction (Figure 2B). Because dye‐derived fluorescent signals were observed to redistribute and transfer between co‐cultured cells over time, double‐positive cells were interpreted as cells acquiring counterpart‐derived fluorescent signals.

Cell ploidy was assessed by propidium iodide (PI) staining. The proportion of 4N cells was 11.30% in MSCs, 8.04% in SFs, and 9.90% in miSF‐MSCs, indicating predominantly diploid DNA content. (Figure S1B). This suggests that nuclear fusion or multinucleation was unlikely to be a major contributor to the double‐positive population. Therefore, the double‐positive cells were interpreted as MSCs acquiring red dye‐labeled SF‐derived fluorescent signals through co‐culture‐dependent material exchange. Morphologically, MSCs were elongated and spindle‐shaped, whereas SFs were shorter. miSF‐MSCs displayed intermediate features and, by passage 3, became more SF‐like (Figure 2C; Figure S2). These observations are consistent with MSC plasticity and microenvironment‐driven phenotypic adaptation [16, 34]. Prior studies similarly demonstrated that co‐culture can redirect MSC transcriptional programs and lineage characteristics, including acquisition of nucleus pulposus‐like [35] or pericyte‐like features [36], with cell–cell contact contributing to these differentiation‐associated changes [13].

### 2.2 Isolation and Characterization of miSF‐MSC‐EVs

**FIGURE 3:**
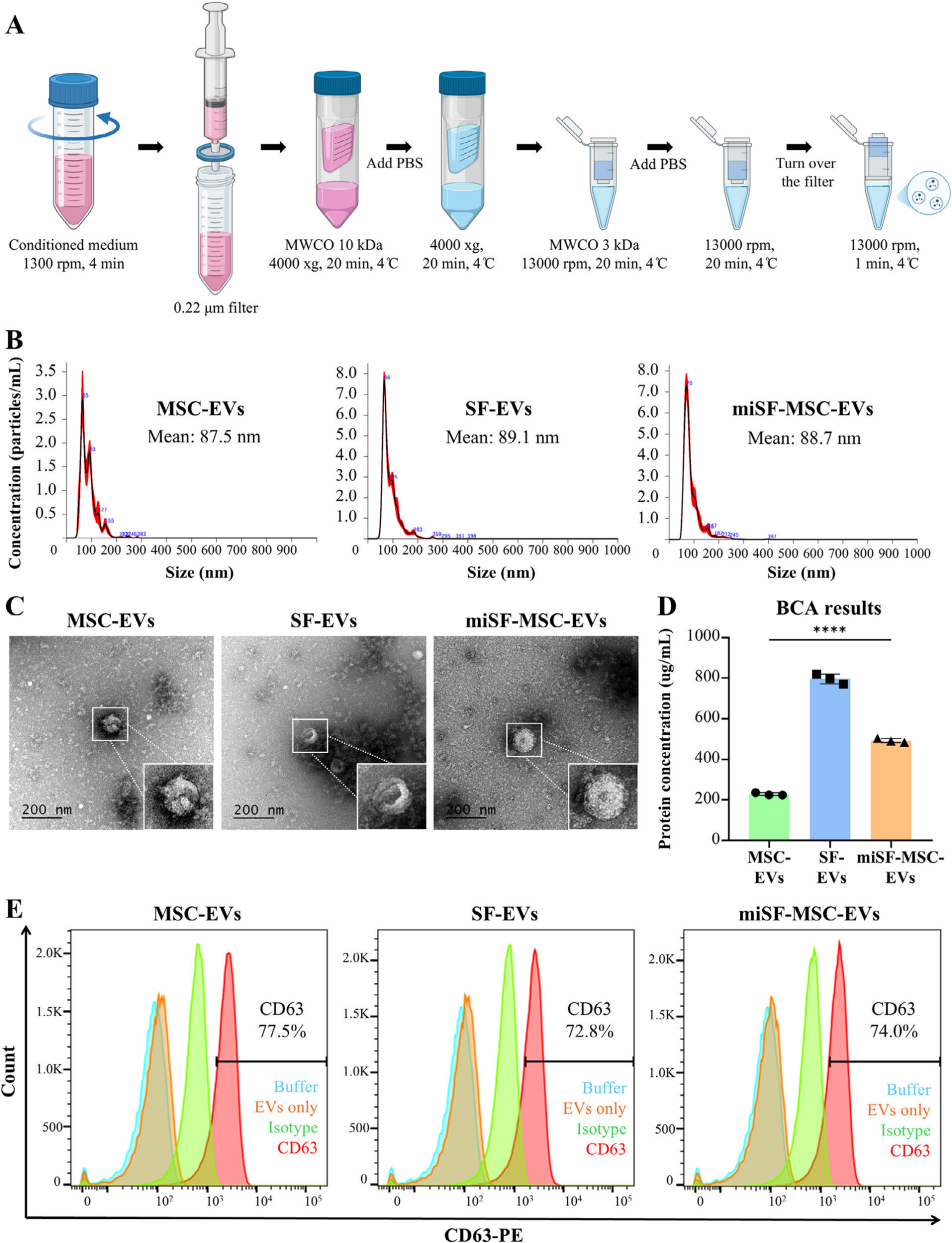
Isolation and characterization of EVs. (A) The process of EV isolation is illustrated as a flow chart. The EVs were isolated using the ultrafiltration (UF) method. (B) The size distribution of EVs was analyzed using NTA. (C) TEM images showed the morphology of EVs. The length of the scale bars: 200 nm. (D) The protein concentration of EVs was measured using the BCA assay. (E) The EV‐positive marker, CD63, was confirmed using flow cytometry. The data are presented as the mean ± S.D. ****p < 0.0001.

EV‐based approaches are increasingly explored for OA because of favorable safety profiles [19]. However, differences in EV isolation methods, including ultracentrifugation, ultrafiltration, size exclusion chromatography, and commercial kits, complicate cross‐study comparisons [37]. Here, miSF‐MSCs were stably expanded to passage 3, and EVs were isolated from conditioned medium by ultrafiltration after cultures reached 80%–90% confluency (Figure 3A).

Nanoparticle tracking analysis (NTA) showed comparable EV sizes across groups: MSC‐EVs, 87.5 ± 0.6 nm; SF‐EVs, 89.1 ± 0.8 nm; and miSF‐MSC‐EVs, 88.7 ± 1.0 nm (Figure 3B). Transmission electron microscopy (TEM) revealed cup‐shaped vesicles consistent with typical EV morphology (Figure 3C). EV protein concentrations measured by BCA assay were 228.1 ± 6.7 µg/mL for MSC‐EVs, 795.8 ± 24.1 µg/mL for SF‐EVs, and 492.5 ± 10.3 µg/mL for miSF‐MSC‐EVs, placing miSF‐MSC‐EVs intermediate between the parental populations (Figure 3D). The higher protein yield of SF‐EVs is consistent with the more rapid proliferation and shorter doubling time of SFs. To further validate EV preparations, vesicles were labeled with a PE‐conjugated anti‐CD63 antibody and quantified by flow cytometry [38]. CD63‐positive events were 77.5% for MSC‐EVs, 72.8% for SF‐EVs, and 74.0% for miSF‐MSC‐EVs (Figure 3E). Together with NTA‐based particle size analysis, TEM‐based vesicular morphology, and CD63 flow cytometry, these results demonstrate successful enrichment of EVs.

### 2.3 Profiling of miSF‐MSC‐EVs with Proteomics and NGS

**FIGURE 4:**
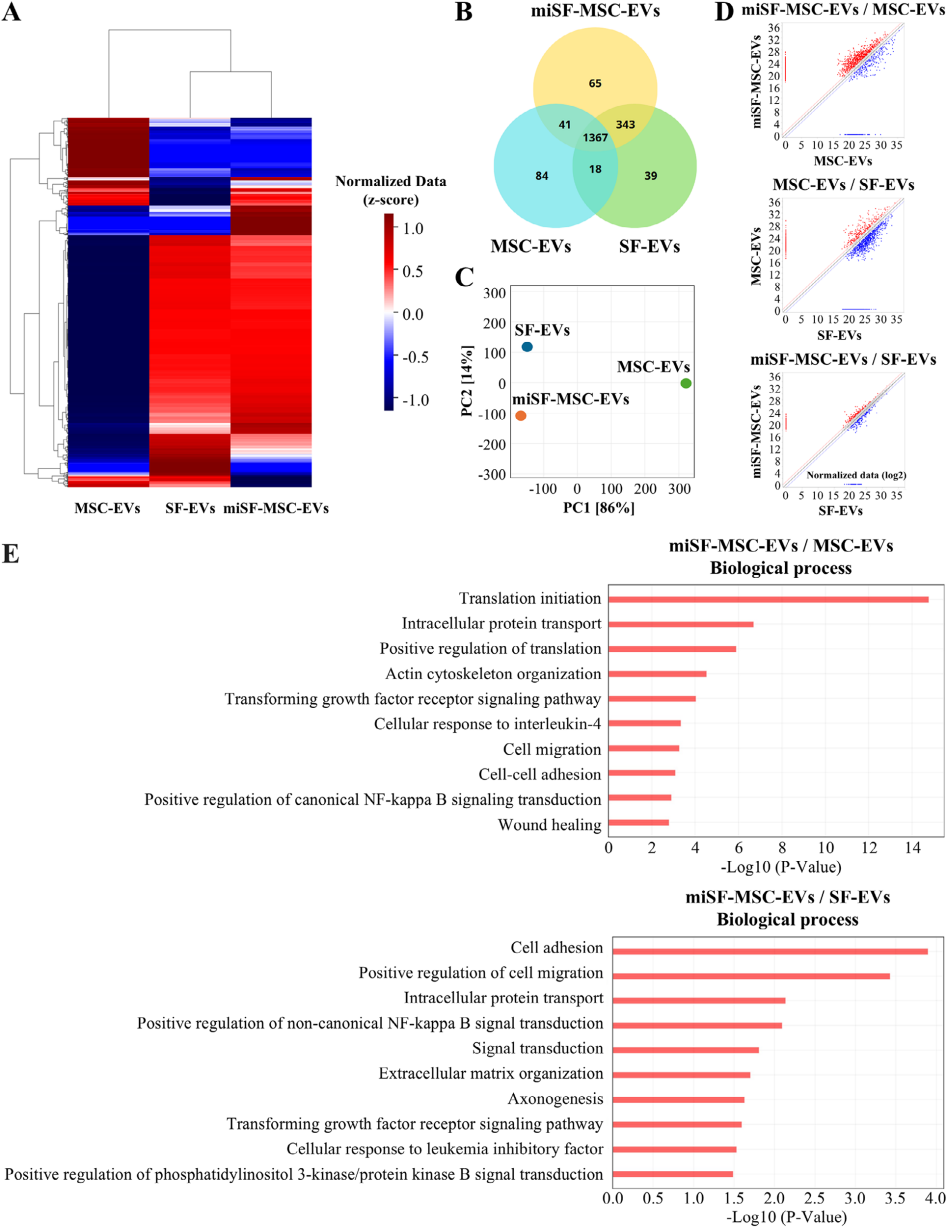
Proteomics analysis of miSF‐MSC‐EVs. (A) The heatmap showed differentially expressed proteins by clustering similar patterns in the normalized data of each EV. (B) The Venn diagram showed differentially expressed proteins between MSC‐EVs, SF‐EVs, and miSF‐MSC‐EVs. (C) The principal component analysis (PCA) showed the clustering patterns of each EV. (D) The scatter plot showed the relationship between miSF‐MSC‐EVs/MSC‐EVs, MSC‐EVs/SF‐EVs, and miSF‐MSC‐EVs/SF‐EVs. (F) Among the proteins with a fold change of 5 or more in miSF‐MSC‐EVs/MSC‐EVs and miSF‐MSC‐EVs/SF‐EVs, biological processes related to tissue regeneration were identified.

Understanding the molecular cargo of EVs is essential for defining how EVs mediate regenerative and immunomodulatory effects and for improving EV‐based therapies [39]. Proteomic (LC–MS/MS) profiling showed that EV composition varied by cell type, with miSF‐MSC‐EVs exhibiting patterns more similar to SF‐EVs than to conventional MSC‐EVs (Figure 4A–D). Based on this proteomic anlaysis, all EVs expressed canornical EV‐associated proteins, including CD63, CD81, ALIX and HSPA8. In contrast, representative Golgi‐, mitochondrial‐, and peroxisome‐associated protein, including GM130, ABCD3, ATP5A1 and TOMM20, were not detected. (Figure S3). Prior proteomic studies have linked MSC‐EVs to biological processes such as cell adhesion and PI3K signaling [40, 41]. In our dataset, these terms were increased by more than fivefold in miSF‐MSC‐EVs relative to MSC‐EVs and SF‐EVs. miSF‐MSC‐EVs were also enriched for processes associated with tissue regeneration, including cell migration, cellular response to IL‐4, wound healing, and TGF‐β signaling (Figure 4E). Collectively, these findings indicate that miSF‐MSC‐EVs carry cargo consistent with enhanced therapeutic activity.

**FIGURE 5:**
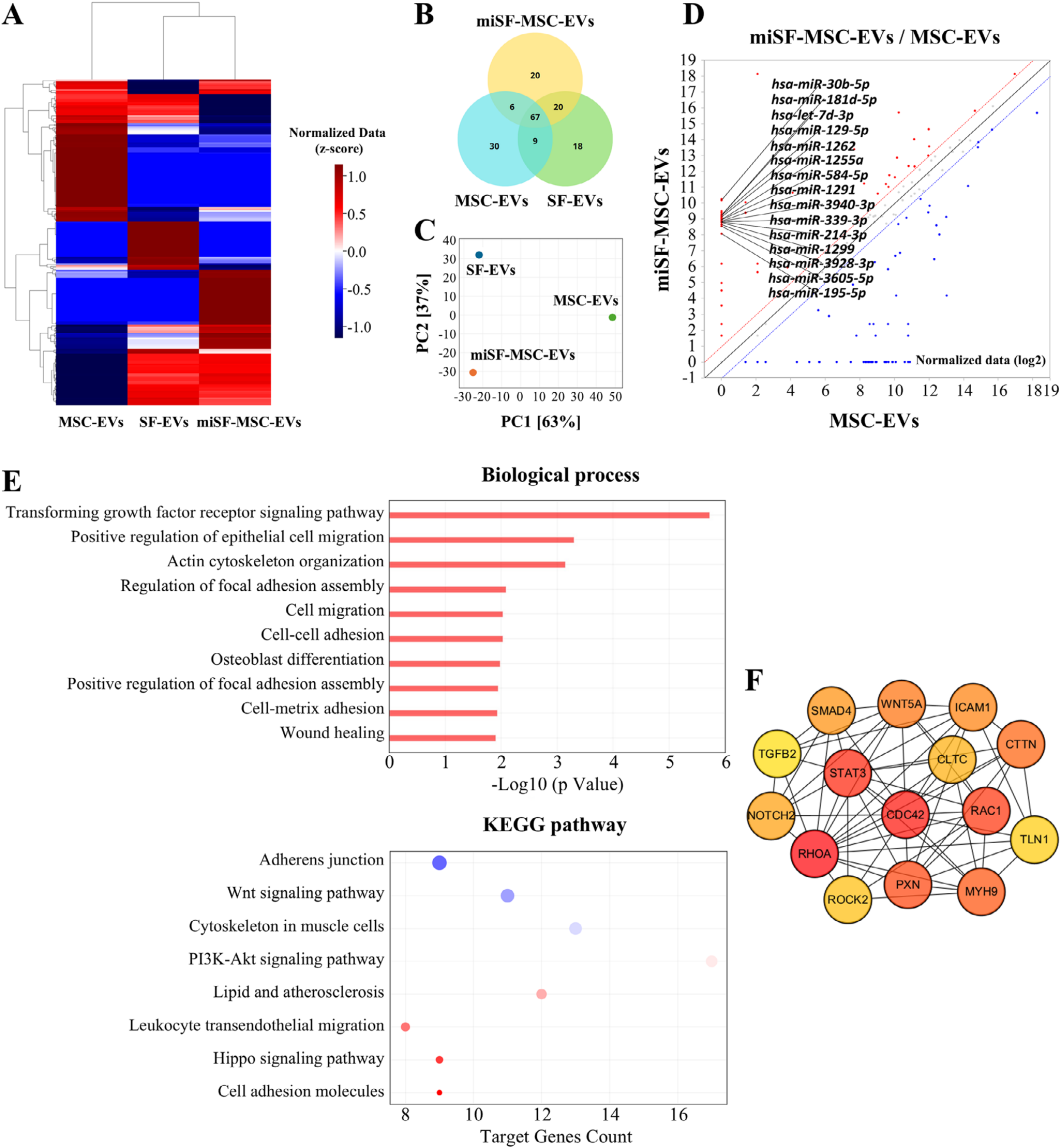
miRNA profiling of miSF‐MSC‐EVs. (A) The heatmap showed differentially expressed miRNAs by clustering similar patterns in the normalized data of each EV. (B) The venn diagram showed differentially expressed miRNAs between MSC‐EVs, SF‐EVs, and miSF‐MSC‐EVs. (C) The principal component analysis (PCA) showed the clustering patterns of each EV. (D) The top 15 specific miRNAs in miSF‐MSC‐EVs were selected from RNA‐seq results. (E) Among the genes with a fold change of 5 or more in miSF‐MSC‐EVs compared to MSC‐EVs, biological processes and KEGG pathways related to tissue regeneration were identified. (F) The network showed interactions between the top 15 hub genes of miRNA target genes that were selected by Cytoscape.

As miRNAs participate in complex regulatory networks, defining the contributions of EV‐derived miRNAs and their targets is critical for clarifying mechanisms and advancing personalized EV‐based therapies [42, 43]. Consistent with this, small RNA‐seq revealed a distinct miRNA profile in miSF‐MSC‐EVs (Figure 5A–C), supporting the concept that direct interaction with SFs reprograms MSC phenotype and gene expression. MSC‐EV‐associated miRNAs have been reported to modulate signaling pathways that promote immunoregulation and chondrocyte proliferation [15, 44, 45]. We therefore focused on the top 15 miRNAs enriched specifically in miSF‐MSC‐EVs (Figure 5D; Table S1). Among these, hsa‐miR‐129‐5p [29], hsa‐miR‐214‐3p [28], and hsa‐miR‐195‐5p [30] have been associated with anti‐inflammatory and/or chondroprotective effects in OA. Gene Ontology (GO) and KEGG analyses of predicted target genes, including those overlapping with the proteomics dataset, indicated enrichment of cell adhesion, cell migration, osteoblast differentiation, and TGF‐β, Wnt, and PI3K signaling relative to MSC‐EVs (Figure 5E). From a protein–protein interaction network constructed using GO‐derived genes, we identified 15 hub genes with high centrality using Cytoscape. These hub genes mapped to TGF‐β, Wnt, Notch, and JAK/STAT signaling, as well as cytoskeletal organization, cell migration, and cell adhesion, with the most significant hub gene highlighted in red (Figure 5F). Together, these analyses support a model in which miSF‐MSC‐EV‐derived miRNAs regulate gene networks associated with cell survival and immunomodulation, consistent with therapeutic potential in OA.

### 2.4 Effects of miSF‐MSC‐EVs on the In Vitro OA Model

We next assessed the regenerative effects of EVs in SFs, human chondrocytes, and human osteoblasts. In the absence of a universally established in vitro EV dose for all recipient cell types, we employed 1 × 10^8^ particles/mL as an intermediate concentration supported by previous studies and used it consistently across all in vitro experiments [46, 47]. Proliferation was quantified by CCK‐8 assays at days 1, 3, and 5, and viability was confirmed by Live/Dead staining at 24 h. In an IL‐1β‐induced in vitro OA model, miSF‐MSC‐EV treatment increased proliferation and viability while reducing cell death (Figure 6A,B). To evaluate EV uptake and cytoskeletal responses, EVs were labeled with a PE‐conjugated anti‐CD63 antibody, and internalization and vinculin expression were examined by confocal laser scanning microscopy after 24 h. All EV preparations were efficiently internalized, and MSC‐EV and miSF‐MSC‐EV treatment increased vinculin expression in SFs, consistent with sequencing results implicating miSF‐MSC‐EVs in cell migration (Figure 6C). Cell migration was further assessed by wound‐healing assays at 12, 24, and 48 h, and miSF‐MSC‐EVs produced the greatest wound closure (Figure S4A,B). As coordinated migration of stem cells, immune cells, and fibroblasts to injury sites is central to tissue repair, these findings support a regenerative phenotype [12].

FIGURE 6Therapeutic effects of miSF‐MSC‐EVs on the in vitro OA model. (A) Cell proliferation was confirmed using CCK‐8 after EV treatment for days 1, 3, and 5 in SFs, chondrocytes, and osteoblasts induced by IL‐1β. (B) Cytotoxicity of EVs was assessed using the Live/Dead assay after EV treatment for 24 h. The length of the scale bars: 100 µm. (C) Intercellular EV uptake and vinculin expression were confirmed using ICC. The length of the scale bars: 10 µm. (D) mRNA expression levels of inflammatory markers after 24 h of EV treatment were analyzed using qPCR. The data are presented as the means ± S.D. (n = 6, biologically independent samples). ns = not significant, *p < 0.05, **p < 0.01, ***p < 0.001, and ****p < 0.0001.

**Figure:**
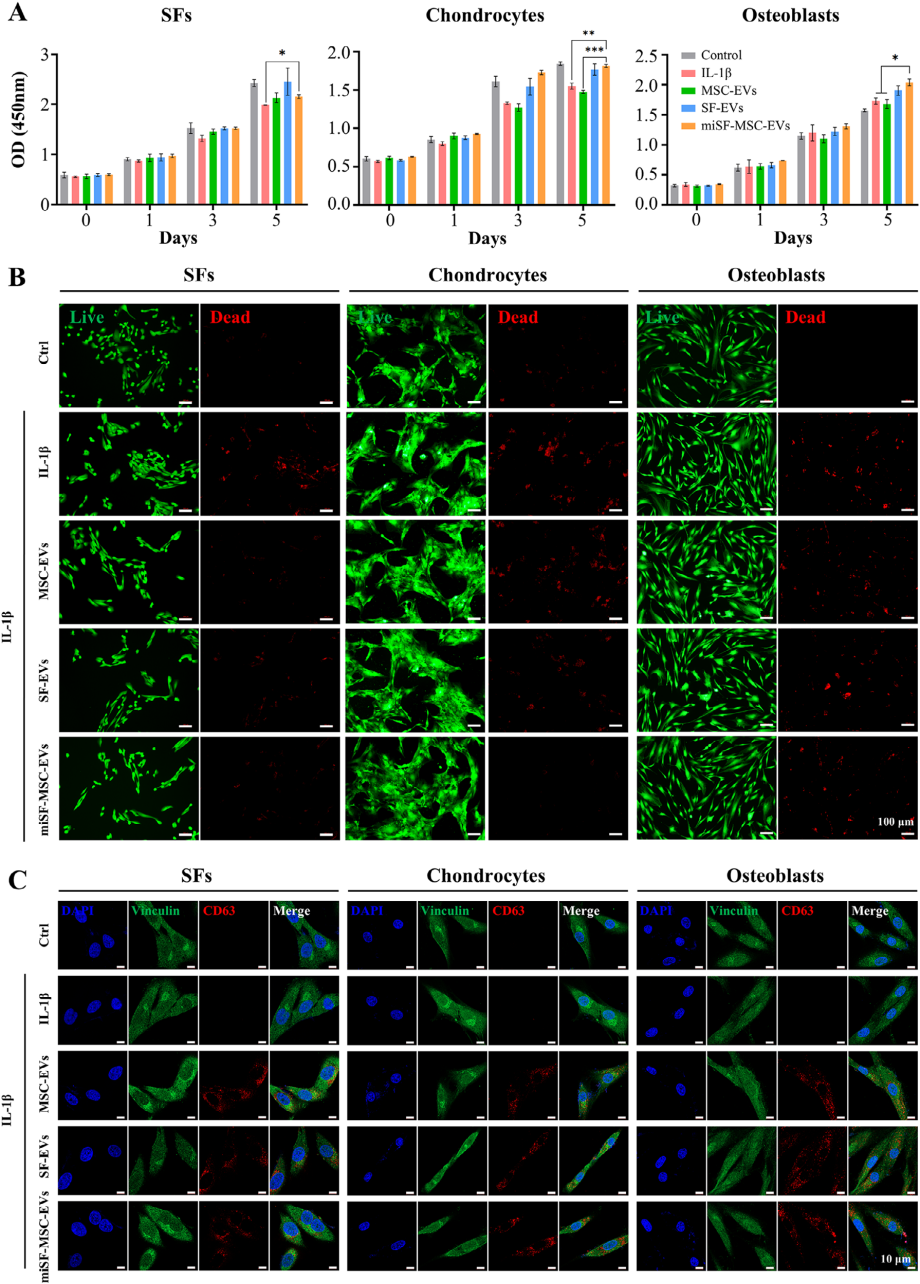

**Figure:**
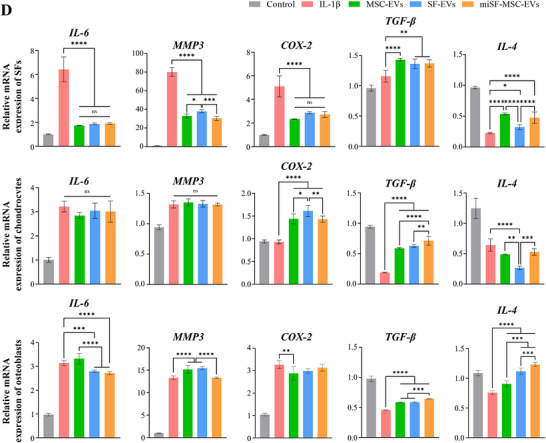

Quantitative PCR showed that miSF‐MSC‐EVs significantly reduced expression of inflammatory markers (IL‐6, MMP3, and COX‐2) in SFs but not in chondrocytes or osteoblasts, suggesting that EV‐mediated immunomodulation differs between immortalized synovial cells and primary cell types [48]. In contrast, expression of the anti‐inflammatory cytokine TGF‐β increased across all cell types (Figure 6D). These results align with sequencing data indicating enrichment of cell adhesion, cell migration, and TGF‐β signaling in miSF‐MSC‐EVs. TGF‐β is a key mediator of tissue regeneration and wound healing, promoting proliferation, differentiation, angiogenesis, and extracellular matrix synthesis while limiting excessive inflammation [49]. Prior studies have shown that TGF‐β‐stimulated MSC‐EVs can enhance chondrocyte proliferation and regeneration through miR‐135b [50] and that delivery strategies incorporating TGF‐β‐enriched MSC‐EVs can provide chondroprotective and anti‐inflammatory effects [51]. Together, these observations underscore the relevance of TGF‐β to OA therapy and support improved efficacy of miSF‐MSC‐EVs relative to conventional MSC‐EVs.

### 2.5 Effects of miSF‐MSC‐EVs on the In Vivo OA Model

**FIGURE 7:**
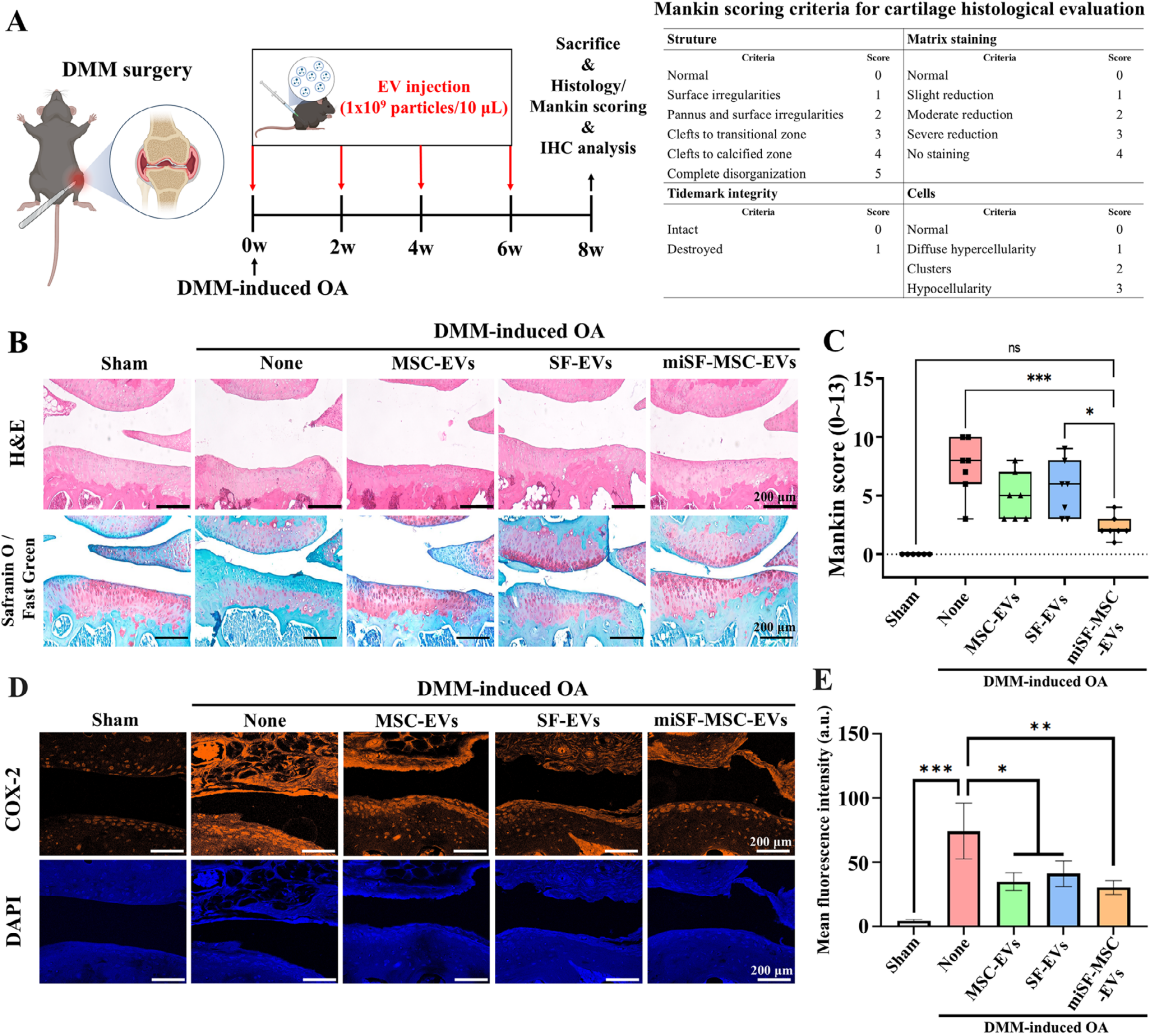
Therapeutic effects of miSF‐MSC‐EVs on the in vivo OA model. (A) Time schedules of in vivo analysis and Mankin scoring criteria. (B) Representative images of the H&E staining and the Safranin O/Fast Green staining. The length of the scale bars: 200 µm. (C) Mankin score evaluation of each group (left) and detailed scoring criteria (right table). The data are presented as the means ± S.D. (Sham n = 6, Others n = 7, biologically independent samples). ns = not significant, ***p < 0.001. (D) Representative immunohistochemical staining of COX‐2 in joint tissues as an inflammation‐associated marker. (E) Quantification of COX‐2 fluorescence intensity in cartilage tissue. The data are presented as the means ± S.D. (n = 3). *p < 0.05, **p < 0.01, and ***p < 0.001.

Finally, we evaluated therapeutic efficacy in a destabilization of the medial meniscus (DMM) mouse model of OA (Figure 7A). Cartilage regeneration was assessed histologically by hematoxylin and eosin (H&E) and Safranin O/Fast Green staining. The None group exhibited marked cell loss and extensive proteoglycan depletion. In contrast, the miSF‐MSC‐EV group showed abundant cellularity and cartilage architecture resembling the sham group (Figure 7B). Cartilage degeneration was quantified using the Mankin scoring system [52], which revealed significantly lower scores in the miSF‐MSC‐EV group than in the None, MSC‐EV, and SF‐EV groups (Figure 7C). These data demonstrate robust cartilage repair and therapeutic benefit of miSF‐MSC‐EVs in vivo. To assess inflammation‐related responses, COX‐2 was examined by IHC. Representative staining images showed increased COX‐2‐positive signals in the None group compared with the sham group, indicating enhanced inflammatory responses in the damaged joint tissues. In contrast, the EV‐treated groups appeared to show reduced COX‐2‐positive staining compared with the None group (Figure 7D). Quantitative data also showed a lower mean fluorescence intensity of COX‐2 in the all EV‐treated groups including miSF‐MSC‐EVs compared with the None groups. These observations suggest that EV treatment may alleviate inflammation‐associated responses in the damaged joint tissues (Figure 7E). Collectively, these results suggest that miSF‐MSC‐EVs exert enhanced therapeutic effects in vivo through both cartilage repair and reduction of OA‐associated inflammation. Additionally, the use of only male mice is a limitation of this study, although this approach was based on previous reports demonstrating a more consistent and pronounced structural OA phenotype after DMM surgery in male mice [53, 54]. Future studies including female mice are warranted to assess sex‐specific treatment responses.

## 3 Conclusions

In summary, miSF‐MSC‐EVs were generated through direct MSC–SF interactions and exhibited molecular signatures consistent with enhanced regenerative and immunomodulatory activity. Proteomic and small RNA‐seq analyses implicated pathways involved in cell adhesion, migration, and TGF‐β signaling, and functional assays demonstrated improved proliferation, migration, and inflammation suppression, particularly in SFs. In the mouse OA model, miSF‐MSC‐EVs enhanced cartilage regeneration and suppressed inflammation, highlighting their efficacy in OA. These findings support miSF‐MSC‐EVs as a promising therapeutic platform for OA. More broadly, this co‐culture approach may be applied to distinct cell lineages to generate cells with reprogrammed gene expression and tailored EV‐mediated effects.

## 4 Experimental Section

### 4.1 Cell Culture

Human bone marrow‐derived mesenchymal stem cells (MSCs; ATCC, USA) and SW982, a human synovial fibroblast‐like cell line (SFs; ATCC), were cultured in high glucose Dulbecco's Modified Eagle's Medium (DMEM) supplemented with 10% (v/v) fetal bovine serum (FBS) and 1% penicillin‐streptomycin (P/S) (GenDEPOT, USA). Primary human chondrocytes (provided by Dongguk University) and primary human osteoblasts (Lonza, Switzerland) were cultured in low glucose DMEM (Gibco, USA) supplemented with 10% FBS and 1% P/S. The medium was changed every 2 days, and the cells were maintained in 5% CO2 at 37°C and were passaged at 80% confluency.

### 4.2 miSF‐MSCs Sorting and Culture

To generate a microenvironment of SF (miSF)‐transferred MSCs (miSF‐MSCs), MSCs and SFs were co‐cultured. They were labeled with NucSpot Live 488 and NucSpot Live 650 (40081, 40082, Biotium, USA) at 1 µL/mL with verapamil at 0.5 µL/mL for 4 h. After detaching the cells with trypsin (GenDEPOT), they were co‐cultured at a 1:1 ratio (30,000 cells/cm^2^) for 48 h. Double‐positive cells were observed by confocal laser scanning microscopy (CLSM; LSM880 with Airyscan, Carl Zeiss, Germany). To obtain miSF‐MSCs, double‐positive cells were isolated from the co‐cultured cells using a flow cytometry sorter (FACSymphony S6, Becton Dickinson, USA). For flow cytometric analysis and sorting, debris and non‐singlet events were excluded by sequential gating based on FSC/SSC and FSC‐A/FSC‐H profiles. Double‐positive events were operationally defined relative to the fluorescence distribution observed in single‐population controls. The sorted miSF‐MSCs were cultured in high glucose DMEM supplemented with 10% v/v FBS and 1% v/v P/S and were used for analyses at passage 3.

### 4.3 Isolation of EVs

Before using the exosome‐depleted FBS (Gibco, USA) in the experiment, centrifugation was performed at 3000 ×g for 55 min at 4°C in Vivaspin Turbo 15 Centrifugal Concentrator Regenerated Cellulose, MWCO 10 kDa (Sartorius, Germany). The ultrafiltered liquid was used to remove remaining extracellular vesicles (EVs) [55]. After reaching 80%–90% confluence, the medium was changed high glucose DMEM supplemented with 10% v/v exosome‐depleted FBS and 1% v/v P/S for 24 h. The medium was harvested and centrifuged at 1300 rpm for 4 min. The supernatant was filtered using a 0.22 µm filter (Sartorius) to remove remaining cells and debris, and then centrifuged twice at 4000 ×g for 20 min at 4°C in Vivaspin Turbo 15. DPBS was used to resuspend the purified EVs. Subsequently, the EVs were collected through centrifugation twice at 13000 rpm for 20 min in Amicon Ultra‐0.5 Centrifugal Filter Unit (Merck Millipore, USA).

### 4.4 Characterization of EVs

The EVs were characterized by morphology, size distribution, and protein concentration using transmission electron microscopy (TEM; CM120, Philips, Netherlands), nanoparticle tracking analysis (NTA; NS300, Malvern, England), and Bicinchoninic acid assay (BCA assay; Pierce BCA Protein Assay Kit, A65453, Thermo Fisher Scientific, USA), respectively. To detect the EV‐specific marker CD63, EVs were incubated with anti‐human CD63‐PE (12‐0639‐41, Invitrogen, USA) or mouse IgG1 kappa isotype control‐PE (12‐4714‐82, Invitrogen, USA) (0.125 µg/test) in the dark for 1 h at 4°C. After washing twice with PBS containing 0.1% BSA at 13,000 ×g for 10 min, EVs were analyzed using flow cytometry (FACSymphony A3, Becton Dickinson). Size calibration was performed using Megamix‐Plus SSC size beads (7803, BioCytex, France). Data from 50,000 gated events were acquired, and the percentage of CD63‐positive EVs was calculated.

### 4.5 Profiling of EVs

Proteomics (LC‐MS/MS) and next‐generation sequencing (NGS; small RNA‐seq) analyses were performed by ebiogen (Korea). Heat maps were generated using MeV, and Venn diagrams were created using the online tool (https://‌bioinformatics.psb.ugent.be/‌webtools/‌Venn/) and visualized using Canva. Principal component analysis (PCA) and scatter plots were generated using ExDEGA. Comparisons of MSC‐EVs and SF‐EVs with miSF‐MSC‐EVs were conducted, and genes with a fold change ≥5 and associated with tissue regeneration were identified using DAVID. The target genes of the top 15 miRNAs that were highly expressed in miSF‐MSC‐EVs were identified using miRTarBase. LC‐MS/MS data were filtered to include only expressed genes, and genes with a fold change ≥5 compared to MSC‐EVs and SF‐EVs were analyzed using Gene Ontology (GO) and KEGG pathway analysis. Highly interacting genes were identified using String, and the top 15 hub genes were visualized with Cytoscape.

### 4.6 Cell Viability and Proliferation

Cell proliferation in response to EV treatment (1 × 10^8^ particles/mL) was examined using a Cell Counting Kit‐8 (CCK‐8) assay (CK04, Dojindo, Japan). A total of 2 × 10^4^ cells were seeded in 24‐well plates and treated with IL‐1β (10 ng/mL; HY‐P7028, MCE) for 24 h, followed by EV treatment every 2 days. The CCK‐8 solution was diluted 1:10 in medium, added to the cells, and incubated for 1 h at 37°C. Absorbance at 450 nm was measured on days 0, 1, 3, and 5. Cell viability was confirmed using a viability kit assay (LIVE/DEAD Cell Imaging Kit (488/570)) (R37601, Invitrogen). A total of 2 × 10^4^ cells were seeded in 24‐well plates and treated with IL‐1β (10 ng/mL) for 24 h, followed by EV treatment (1 × 10^8^ particles/mL) for 24 h. After washing with PBS, the cells were incubated with a mixture of green (live) and red (dead) solutions for 15 min at room temperature (RT) and visualized using a fluorescence microscope. Live cells showed green fluorescence due to Calcein AM staining, and dead cells showed red fluorescence due to BOBO‐3 iodine staining. EV concentration was selected as a literature‐supported intermediate dose within the commonly used in vitro EV dosing range and was used consistently across SFs, chondrocytes, and osteoblasts [46, 47].

### 4.7 Immunocytochemistry

A total of 1.5 × 10^4^ cells were seeded in a confocal plate (211350, SPL) and treated with IL‐1β (10 ng/mL) for 24 h. EVs were incubated with anti‐human CD63‐PE (0.125 µg/test) in the dark for 1 h at 4°C. After washing twice with PBS containing 0.1% BSA at 13,000 rpm for 10 min, EVs were incubated with the cells for 24 h. Cells were then fixed with 4% paraformaldehyde (Biosesang, Korea), permeabilized with 0.05% Triton X‐100 (Sigma, USA), and blocked with 2% BSA in PBS for 1 h at RT. Subsequently, the samples were incubated with anti‐vinculin primary polyclonal antibody (1:500 in PBS with 0.1% BSA; PA5‐29688, Invitrogen) overnight at 4°C, followed by incubation with an Alexa Fluor 594 Goat Anti‐Rabbit IgG secondary antibody (1:1000; ab150080, Abcam, UK) for 45 min at RT. Nuclei were stained with DAPI (2 µg/mL) for 5 min at RT.

### 4.8 RNA Isolation and RT‐qPCR

SFs, chondrocytes, and osteoblasts were treated with IL‐1β (10 ng/mL) for 24 h, followed by treatment with EVs at a concentration of 1 × 10^8^ particles/mL for 24 h. Total RNA was extracted from cells using TRIzol reagent (15596018, Invitrogen). Complementary DNA (cDNA) was synthesized using the cDNA Synthesis kit (Bio‐Rad, USA), and RNA concentration was quantified with a Nanodrop Lite spectrophotometer (Thermo Fisher Scientific). Quantitative real‐time PCR (qPCR) was performed in triplicate using the TOP real qPCR 2X PreMIX (SYBR Green with low ROX) (Enzynomics, Korea) on a Quant Studio 1 (Thermo Fisher Scientific) according to the manufacturer's instructions. Relative gene expression levels were calculated using the 2‐ΔΔCt method. The primer sequences used for the qPCR analysis were listed in Table S2.

### 4.9 OA Induction and Treatments

All male C57BL/6J mice (8 weeks old, weighing 18–24 g) were provided by SAMTAKO BIO KOREA (Osan, Korea). Male mice were used to ensure a stable post‐traumatic OA model, as previous studies have reported that DMM‐induced cartilage degenration and disease severity was influenced by sex and was often more pronounced in male mice [53, 54]. Mice were anesthetized with the mixture of tiletamine hydrochloride and zolazepam hydrochloride (Zoletil, 50 mg/kg, Virbac Laboratories, France) and xylazine (Rompun, 10 mg/kg, Bayer, Korea). OA was induced by dissecting the medial meniscus ligament to destabilize the medial meniscus in the right knee joint of the hind limb, as described previously in the DMM model [56]. Randomly, the mice were divided into five groups: Group 1 (n = 6) underwent knee joint exposure only. They were surgically treated in the same manner except for not dissecting the medial meniscus ligament. (Sham operation); Groups 2–5 (n = 28) underwent DMM to the knee joints. After surgery, the animals were allowed to fully recover from the anesthesia and were monitored appropriately before returning them to the cages. For postoperative analgesia, ketoprofen was administered subcutaneously at 5.0 mg/kg once daily for 3 days. The mice in groups 2–5 were intra‐articularly injected with PBS, MSC‐EVs, SF‐EVs, and miSF‐MSC‐EVs, respectively. EVs (1 × 10^9^ particles/10 µL) were injected every 0, 2, 4, and 6 weeks, and the animals were sacrificed at 8 weeks. The mice were housed in a conventional animal facility under controlled conditions (22 ± 2°C, 50 ± 10% humidity, 12 h light/dark cycle), with three mice per cage and free access to standard chow and water. All animal care and experimental procedures were performed in accordance with the Institutional Animal Care and Use Committee (IACUC) of the Center for Laboratory Animal, Atems Co., Ltd. (ATEMS‐IACUC‐2025‐004).

### 4.10 Histologal Evaluation

The right knee joints of the mice in various groups were obtained and fixed in 10% formalin for 72 h, then decalcified in 10% EDTA for 4 weeks and embedded in paraffin. Histomorphometric changes were assessed by microscopic examination of serial 4 µm‐thick longitudinally oriented sections. The samples were stained with safranin O/fast green according to the manufacturer's instructions and were scored using a modified Mankin score (Figure 7C) (score range: 0–13, from normal to most severe reaction) [52]. Fluorescent immunostaining for COX‐2 was additionally performed on paraffin‐embedded knee joint sections. After deparaffinization, rehydration, and antigen retrieval, the sections were incubated with an anti‐COX‐2 primary antibody (Cat. No. AMRe01846, EnkiLife), followed by an appropriate fluorescent secondary antibody (Cat. No. bs‐0295D‐BF555, Bioss). The stained sections were observed under a fluorescence microscope. Quantification was performed from fluorescence images using ImageJ software. Mean fluorescence intensity was measured from defined cartilage ROIs after excluding tissue‐free areas.

### 4.11 Statistical Analysis

The data were presented as the means ± S.D. (Standard Deviation). All statistical analyses were performed using GraphPad Prism 8 software (La Jolla, CA, USA). The one‐way ANOVA was used to compare multiple groups, and the outcomes were analyzed using a Tukey post hoc test for statistical analysis. *p* < 0.05 was considered statistically significant.

## Funding

This research was supported by the National Research Foundation of Korea (NRF) grant funded by the Korea government (MSIT) (RS‐2023‐00277856) and grant of the Korea Health Technology R&D Project through the Korea Health Industry Development Institute (KHIDI), funded by the Ministry of Health & Welfare, Republic of Korea (RS‐2025‐24535069).

## Conflicts of Interest

The authors declare no conflicts of interest.

## Supporting information




**Supporting File**: adhm71406‐sup‐0001‐SuppMat.docx.

## Data Availability

The data that support the findings of this study are available from the corresponding author upon reasonable request.
