# Supplementary material for: Therapeutic Extracellular Vesicles from Synovial Fibroblast‐Primed MSCs for Osteoarthritis Treatment
Source: Adv Healthc Mater. 2026 Jul 6;15(30):e71406. doi: 10.1002/adhm.71406 (PMC13474136; doi:10.1002/adhm.71406)
Supplement: Supplementary file 1 [file ADHM-15-0-s001.docx]

Copyright WILEY-VCH Verlag GmbH & Co. KGaA, 69469 Weinheim, Germany, 2019.

Supporting Information

Therapeutic Extracellular Vesicles From Synovial Fibroblast-Primed MSCs for Osteoarthritis Treatment

Seo Jeong Kim^§^, Ji Seob Kim, Min Hee Moon, Jae-Hyeok Jang, In Sun Hwang, Byung-Hyun Cha*

**Experimental Section**

*Ploidy analysis:* The ploidy of MSCs, SFs, and miSF-MSCs was analyzed by PI staining. The cells were seeded in 6-well plates at a density of 1 × 10^5^ cells per well and cultured until confluence. After cells were detached by trypsin, they were fixed in 66% ethanol overnight at 4 °C. The samples were resuspended and centrifuged at 500 xg for 5 min. After PBS washing, they were incubated with RNase followed by PI staining (ab139418, Abcam) for 30 min at 37 °C. Flow cytometry analysis was performed on gated single cells (debris excluded), acquiring data from 10,000 gated events per sample. Ploidy distribution was calculated as percentages of each population.

*Wound Healing Assay:* SFs were cultured in 6-well plates at a density of 3 × 10^5^ cells per well until a confluent monolayer formed and treated with IL-1β (10 ng/mL) for 24 h. To confirm only cell migration, cells were treated with mitomycin C (10 μg/mL; M5353, Sigma) for 1.5 hr in medium containing 5% FBS and then washed twice with PBS. Scratches were made using the SPL Scar™ Scratcher (201907, SPL). After rinsing with PBS solution, EVs were treated at a concentration of 1 × 10^8^ particles/mL for 24 h. Wound healing activity was monitored 12, 24, and 48 h later using microscopy. The percentages of open area were calculated using the wound healing tool plugin in ImageJ (Wayne Rasband, USA).

**Supplementary Figures**

**Figure S1. miSF-MSCs generation and ploidy analysis.** A) Nuclear dyes were transferred during miSF-MSC formation. Red dye was translocated from SF nuclei to the cytosol at 24 and 48 h. The length of the scale bars: 20 μm. B) PI staining revealed normal diploid DNA content in MSCs, SFs, and miSF-MSCs, confirming genomic stability.

**Figure S2. The morphology of miSF-MSCs at passage 2, 3, and 4.** miSF-MSCs showed stable proliferation from passage 3 with SF-like morphology. The length of the scale bars: 100 μm.

**Figure S3. Proteomics analysis of EV markers.** Proteomics data revealed high expression of positive EV markers CD63, CD81, ALIX and HSPA8, with the absence of negative organelle markers GM130, ABCD3, ATP5A1 and TOMM20 in purified EVs.

**Figure S4. The wound healing assay was confirmed after EV treatment in SFs.** A) The wound healing assay was confirmed after EV treatment for 12, 24, and 48 h in SFs. The length of the scale bars: 100 μm. B) The miSF-MSC-EVs group showed the highest rate of wound closure. The data are presented as the means ± S.D. ns = not significant, * *p* <0.05, ** *p* <0.01, *** *p* <0.001, and *****p* <0.000.

**Supplementary Table**

**Table S1.** Selected miRNAs differentially regulated in EVs.

| **miRNA** | **Fold change** | | |
| --- | --- | --- | --- |
|  | **MSC-EVs** | **SF-EVs** | **miSF-MSC-EVs** |
| hsa-miR-30b-5p | 0.000 | 0.000 | 10.174 |
| hsa-miR-181d-5p | 0.000 | 0.000 | 9.233 |
| hsa-let-7d-3p | 0.000 | 0.000 | 9.223 |
| hsa-miR-129-5p | 0.000 | 0.000 | 9.217 |
| hsa-miR-1262 | 0.000 | 0.000 | 9.115 |
| hsa-miR-1255a | 0.000 | 0.000 | 8.949 |
| hsa-miR-584-5p | 0.000 | 0.000 | 8.936 |
| hsa-miR-1291 | 0.000 | 0.000 | 8.851 |
| hsa-miR-3940-3p | 0.000 | 0.000 | 8.844 |
| hsa-miR-339-3p | 0.000 | 0.000 | 8.810 |
| hsa-miR-214-3p | 0.000 | 0.000 | 8.803 |
| hsa-miR-1299 | 0.000 | 0.000 | 8.775 |
| hsa-miR-3928-3p | 0.000 | 0.000 | 8.641 |
| hsa-miR-3605-5p | 0.000 | 0.000 | 8.537 |
| hsa-miR-195-5p | 0.000 | 0.000 | 8.066 |

**Table S2.** Primers used in qRT-PCR.

| **Gene** | **Forward primer (5'→3')** | **Reverse primer (5'→3')** |
| --- | --- | --- |
| *GAPDH* | ACA TCG CTC AGA CAC CAT G | TGT AGT TGA GGT CAA TGA AGG G |
| *IL-6* | AGA CAG CCA CTC ACC TCT TCA G | TTC TGC CAG TGC CTC TTT GCT G |
| *MMP3* | CAC TCA CAG ACC TGA CTC GG | GAG TCA GGG GGA GGT CCA TA |
| *COX-2* | CTC CCT TGG GTG TCA AAG GTA A | GTG CTG GGC AAA GAA TGC AA |
| *TGF-β* | GCA ACA ATT CCT GGC GAT ACC | ATT TCC CCT CCA CGG CTC AA |
| *IL-4* | CCG TAA CAG ACA TCT TTG CTG CC | GAG TGT CCT TCT CAT GGT GGC T |
